# ALVEOLAR BONE PATTERN AND SALIVARY LEPTIN LEVELS AMONG PREMENOPAUSAL OBESE WOMEN

**DOI:** 10.1590/0102-672020180001e1422

**Published:** 2019-02-07

**Authors:** Silvia Helena de Carvalho SALES-PERES, Francisco Carlos GROPPO, Rafaela Carolina Soares BONATO, Matheus de Carvalho SALES-PERES, Francisco HAITER-NETO, Elinton Adami CHAIM

**Affiliations:** 1 Department of Pediatric Dentistry, Orthodontics and Public Health, Bauru School of Dentistry, University of São Paulo, Bauru, SP; 2 Department of Physiological Sciences, Piracicaba Dental School, University of Campinas, Piracicaba-SP;; 3 Department of Surgery, Botucatu Medicine School, University of São Paulo State;; 4 Department of Oral Diagnosis, Division of Oral Radiology, Piracicaba Dental School, University of Campinas, Piracicaba, SP;; 5 Department of Surgery, Faculty of Medical Sciences, University of Campinas, Campinas); Brazil.

**Keywords:** Obesit, Quality of lif, Lepti, Periodontal disease, Alveolar bone loss, Obesidad, Qualidade de vid, Leptin, Doenças periodontai, Perda óssea alveolar

## Abstract

**Background:**

Systemic bone loss may lead to more severe periodontal destruction, decreasing local bone mineral density.

**Aim:**

A cross-sectional designed was performed to study associations among alveolar bone pattern, salivary leptin concentrations, and clinical periodontal status in premenopausal obese and eutrophic women.

**Methods:**

Thirty morbid obese (G1) and 30 normal-weight (G2) women were included. Anthropometric and periodontal measurements (probing depth - PD, clinical attachment levels - CAL, presence of calculus, bleeding on probing -BOP, and plaque accumulation) were assessed. OHIP-14 was used for assessment of oral health impact on quality of life. Panoramic radiography was used to obtain the panoramic mandibular index (PMI), mandibular cortical index (MCI), and mental index (MI). Intraoral periapical (PA) radiography was taken to measure the total trabecular bone volume. Leptin was measured in saliva of fasted overnight women.

**Results:**

Groups 1 and 2 differed in all anthropometric aspects, but height. Pocket depth, calculus, BOP, and plaque index were worse in G1. No differences between groups were found considering OHIP. Normal-weight subjects showed higher proportion of dense bone trabeculae than obese subjects for pre-molars, but not for molars. Mental and panoramic mandibular indexes did not differ and were in normal level. Leptin concentration was dependent only on BMI.

**Conclusion:**

Obesity affected the periodontal conditions, the alveolar bone pattern, and the salivary leptin concentration.

## RESUMO

**Racional:**

A perda óssea sistêmica pode levar à destruição periodontal mais severa, diminuindo a densidade mineral óssea local.

**Objetivo:**

Investigar as associações entre padrão ósseo alveolar, concentrações de leptina salivar e estado periodontal em mulheres obesas na pré-menopausa e eutróficas.

**Métodos:**

Foram avaliadas 30 mulheres com obesidade mórbida (G1) e 30 com peso normal (G2). Foram analisadas as medidas antropométricas e periodontais (profundidade de sondagem - PS, nível clínico de inserção - NCI, presença de cálculo, sangramento à sondagem - SS e acúmulo de placa). O impacto da saúde bucal na qualidade de vida foi mensurado por meio do questionário OHIP-14. Radiografia panorâmica foi utilizada para obter o índice mandibular panorâmico (PMI), índice cortical mandibular (MCI) e índice mental (MI); já a radiografia periapical intraoral (AF) para medir o volume ósseo trabecular total. A leptina salivar foi coletada no período da manhã com a paciente em jejum.

**Resultados:**

Os grupos 1 e 2 diferiram em todos os aspectos antropométricos, exceto em estatura. Profundidade de bolsa, cálculo, SS e índice de placa foram piores no G1. Não foram encontradas diferenças entre os grupos considerando o OHIP. Indivíduos com peso normal apresentaram maior proporção de trabéculas ósseas densas do que os obesos para pré-molares, mas não para molares. Índices radiomorfométricos não diferiram entre os grupos e estavam dentro de valores normais. A concentração de leptina esteve associada ao IMC.

**Conclusão:**

A obesidade afetou as condições periodontais, o padrão ósseo alveolar e a concentração de leptina salivar.

## INTRODUCTION

The association between obesity and periodontitis, and the increased prevalence, severity and progression of periodontitis in obese subjects, has been demonstrated in the literature^1^. Both obesity and periodontitis are associated with systemic inflammation, and it is possible that they are linked by a common pathophysiology. Increasing adipose tissue results in activation of the inflammatory host response, which could increases the susceptibility of obese-subjects to periodontal diseases^2^. However, the relationship between the inflammation observed in both obesity and periodontitis is still unclear. 

Obesity is also related with the type 2 diabetes mellitus (T2DM) development. In turn, T2DM patients have shown increasing propensity to bone fractures, probably due their increased cortical porosity^**3**^. Nevertheless, a high turnover on mineral constituents is usually responsible for bone-cortical porosity, but T2DM patients have reduced bone turnover^4^.

Changes in the immune system promoting inflammatory events could explain the clinical association among obesity, T2DM, and periodontitis. One of the keys for the association could be the adipokines, mainly adiponectin and leptin^5^

The leptin is considered a link between the neuroendocrine and immune systems. It could regulate many physiological and pathophysiological processes, especially inflammation and immunity. Leptin and adiponectin may also regulate bone metabolism and be involved in osteoporosis pathophysiology. High levels of leptin, usually associated with obesity, are risk factor for bone loss^6^.

In high levels, pro-inflammatory cytokines also have osteoclastic effect^7^. Pro-inflammatory cytokines produced by adipose tissue, such as interleukin 6 (IL-6) or tumor necrosis factor α (TNF-α), stimulate osteoclastogenesis^8^. Thus, inflammation may participate in bone loss in obese patients. As previously observed, patients with elevated fat mass, regardless of body weight, had high risk of osteopenia, osteoporosis, and non-vertebral fractures^9^. Postmenopausal osteoporosis is closely associated with estrogen deficiency^10^, and it includes periodontal manifestations, such as alveolar bone resorption, clinical attachment loss and tooth loss^11^.

The hypothesis in the present study was a possible association among alveolar bone pattern, salivary leptin concentrations, and clinical periodontal status in premenopausal obese women. Therefore, the aim of the present study was to compare these variables between morbid-obese and eutrophic premenopausal women. 

## METHODS

This investigation was approved by the Research Ethics Committee of the Amaral Carvalho Hospital and was conducted in full accordance with the World Medical Association Declaration of Helsinki. All research participants signed consent prior to evaluation and all consents were referred to the committee.

### Type, study location and setting

The STROBE guidelines were used to report this cross-sectional study.

It was conducted at a public hospital of Jau City, SP, Brazil, from October 2012 until December 2013. Candidates to bariatric surgery were recruited and examined prior to the initiation of their clinical treatment (n=60). 

### Sample 

The sample size calculation was performed using the difference between two proportions, with drawing effect of 5% and 80% power. Considering a minimum difference to be detected of 2% in the variable “% of sites with 4-5 mm pocket” (largest sample size), and an estimated prevalence of periodontal pockets of 50%, the sample size calculation was 24 subjects per group, according to the design effect. 

### Eligibility criteria

Eligibility criteria sample for obese group consisted of morbid obese patient indicated for bariatric surgery (BMI exceeding 40 kg/m^2^ or ≥35 kg/m^2^ with serious comorbidity), which were treated at Public Health System. Control group was composed by patients with normal weight (BMI ranging 18.50 to 24.99 kg/m^2^) that were attended in the same hospital. Inclusion criteria for both groups were good general health, not using anti-inflammatory or antibiotics drugs, and diagnosed with chronic periodontitis or aggressive periodontitis. Exclusion criteria included history of systemic infection diseases, current pregnancy or lactation, hypertension, sleep disturbances, depression, abuse of alcohol, and individuals with fewer than six teeth. Both groups were matched for age and education.

### Clinical examination 

Individuals were considered obese when their BMI was ≥40 kg/m², and normal weight when BMI varied from 18.5 kg/m² to 24.9 kg/m² ^12^. Clinical examinations were conducted by a previously calibrated dentist (kappa >0.83). All teeth were examined, measuring probing depth (PD), clinical attachment levels (CAL), presence of calculus, and bleeding on probing (BOP). Periodontitis was considered when sites showed PD ≥4 mm and clinical attachment loss ≥3 mm. BOP indicates the presence of gingivitis. 

Plaque accumulation was assessed by the index described by Turesky et al.^13^ which the codes ranging from 0 (no plaque) to 5 (covering ≥2/3 of the area). 

### Oral health impact profile (OHIP-14 questionnaire) 

The Oral Health Impact Profile (OHIP-14) was used for assessment of oral health impact on quality of life and the reference period used was one year^14^. Items are distributed among seven dimensions of impacts of oral conditions including functional limitation, physical pain, psychological discomfort, physical disability, psychological disability, social disability, and deficit. 

### Image assessment

#### Panoramic radiographic examination

The patients mandible was examined on panoramic images performed using a Kodak 8000C unit (Panoramic and Cephalometric Digital System; Kodak, Trophy SAS, France) with a Charge Couple Device Sensor, 90 KVp, 15 mA, and 1.27 magnification. Images with poor quality were excluded from the sample with basis on previously defined criteria as follows: patient positioning errors, faults in density, contrast or detail^15^.

#### Panoramic mandibular index (PMI)

The ratio of the mandibular cortical thickness was measured on the line perpendicular to the bottom of the mandible, at the middle of the mental foramen, by the distance between the inferior mandibular cortex and the bottom of the mandible, as previously described by Benson et al.^16^, considering values ≥0.3 as the normal ratio.

#### Mandibular cortical index (MCI)

Mandibular cortical shapes in the panoramic radiographs were obtained by observing the mandibles distally from the mental foramina in both sides. They were categorized into one of the three groups according to Klemetti et al.^17^: C1 - Normal cortex; C2 - mild to moderately eroded cortex or it appeared to form endosteal cortical residues; and C3 - severely eroded cortex or the cortical layer formed heavy endosteal cortical residues and it was clearly porous.

#### Mental index (MI)

The measurement of the bone-cortical width at the mental foramen region was performed according to the method previously described by Ledgerton et al.^18^. A parallel line from the long axis of the mandible, tangent to the inferior border of the mandible was drawn. A perpendicular line to this tangent intersecting the inferior border of the mental foramen was drawn and the mandibular cortical width was than measured. The mental index (MI) was obtained from the mean mandibular cortical width.

All the linear measurements were performed using the software ImageJ 1.42 k (with Java 1.6.0.05), which provided a magnification correction of 30% to simulate clinical situation.

The radiographs were classified according to the mandibular cortical index (MCI)^17^, considering a qualitative classification of the endosteal margin of the mandibular cortex as follows: C1 - normal; C2 - osteopenia; and C3 - osteoporosis. Patients were classified according to the WHO criteria^10^, i.e., a t-score ranging between -1 and -2.5 standard deviation for osteopenia; and -2.5 standard deviation below the bone mass peak for osteoporosis. When discrepancies in the bone densitometry of spine and femur were verified, the worst classification was considered.

#### Periapical (PA) radiographic assessment

The intraoral periapical (PA) radiographic images were taken with Kodak 6100 (Carestream, Atlanta, GA) using paralleling devices (Dentsply Rinn, Elgin, IL), at 65 kVp, 7 mA, and 0.14 seconds. After optimizing contrast, all scans were aligned to the axes of the apical third. For the axial, sagittal, and coronal planes, the individual slice showing the periodontal ligament space at its largest was identified. Slices were maximized, and a 1-mm scale was placed in the lower right corner of the image before a screenshot was exported in TIFF format. All information that could identify the patient was removed^19^. 

#### Measurement of the total trabecular bone volume

The image was enlarged as much as possible to increase the accuracy when separating the trabecular and cortical bone components. The contrast and brightness of the radiograph were set to give the best possible image of the trabeculae.

For linear measurement of the images (using ImageJ), the long axis of the tooth was traced and subsequently lines passing by the cement-enamel junction (CEJ) at the most coronal bone level next to the tooth and the root apex. All lines should be perpendicular to the line drawn in the long axis of the tooth. The distance of the CEJ was measured until the bone level (CEJ-BL), and the distance to the apex of the JCE (JCE-AP).

The following relation [(^JCE-BL^/_JCE-AP_) × 100] was obtained at the distal aspect of first premolar (34 and 44), mesial and distal of the second premolar (35 and 45) and mesial of the first molar (36 and 46). Bone loss was considered when the result exceeded 10%.

The trabeculation of alveolar bone was evaluated by using the following three-scale visual analysis: score 1 - sparse trabecular pattern; score 2 - dense and sparse trabecular pattern; and score 3 - dense trabecular pattern ^20^.

### Salivary leptin 

Saliva samples were collected the morning after patients had fasted overnight. They were asked to avoid tooth brushing and drinking anything in the morning except water. 

Whole unstimulated salivary samples (2 ml) were collected by a modified draining method^21^. Patients were asked to expectorate into salivette tubes every 30 s over a period of 5 min. Two milliliters of saliva were pipetted into an Eppendorf tube. Saliva samples were centrifuged to remove cell debris, and 0.5 ml of the supernatant was identified, and stored in 1.5-ml aliquots at -80^o^ C until analysis. 

A high sensitive enzyme-linked immunosorbent assay Quantikine® ELISA - Human Leptin Imunoassay (R&DSystems) was used to detect leptin concentrations in saliva. The kit used biotin antibody, horseradish peroxidase-streptavidin solution, and tetramethylbenzidine substrate. Absorbance of the substrate color reaction was read on an ELISA reader (Life Science, Bio-Rad Laboratories, Bio-Rad.com). 

### Statistical analyses

All statistical analyses were carried out by Systat 13.0, BioEstat 5.0, and GraphPad Prism 7.0. Shapiro-Wilk´s and Levene´s tests were used to observe data normality and homoscedasticity of variances, respectively. A significance level of 5% was set for all tests.

Comparisons between normal weight and obese subjects regarding demographics were performed by Mann-Whitney test (age, weight, height, BMI) or unpaired t test with Welch correction (waist circumference, hip and waist-hip ratio). The waist-hip ratio risk was observed by Chi-square test. Both periodontal and OHIP aspects were evaluated by the Mann-Whitney test. This test also compared the distances between the cement enamel junction to the bone level (CEJ-BL) and to the apex (CEJ-AP), alveolar bone loss, and the mental and panoramic mandibular indexes of both groups. Chi-square or Fisher Exact’s tests were used to compare the scores of trabecular bone patterns between groups. The leptin concentration in saliva between groups was compared by unpaired t test. Possible correlation between leptin concentrations and demographic characteristics, the periodontal aspects, OHIP, trabecular bone patterns, and mental and panoramic mandibular indexes were observed by Spearman’s test. A linear regression observed the dependency of leptin concentrations with the same variables.

## RESULTS

Both groups differed in all anthropometric aspects, but height (Table 1). Thus, the main component of BMI was the body weight. Waist-hip ratio risk was significantly higher in obese than in normal-weight women.

**TABLE 1:** Anthropometrics measurements of both groups

Variable		Normal weight (n=30)	Obese (n=30)	p	test
Age (years)	median (interquartile deviation)	24 (4)	31 (5.5)	< 0.0001	Mann-Whitney
Waist circumference (in cm)	mean (±SD)	73.8 (±5.3)	117.6 (±14.8)	< 0.0001	Unpaired t test with Welch correction
Hip (cm)	mean (±SD)	100.6 (±5.7)	144.4 (±15.1)	< 0.0001	Unpaired t test with Welch correction
Waist-Hip ratio	mean (±SD)	0.73 (±0.04)	0.82 (±0.06)	< 0.0001	Unpaired t test with Welch correction
Weight (kg)	median (interquartile deviation)	58 (9)	122.5 (36.5)	< 0.0001	Mann-Whitney
Height (meters)	median (interquartile deviation)	165 (7)	165 (6.8)	0.4025	Mann-Whitney
BMI (kg/m2)	median (interquartile deviation)	21.1 (2.5)	48.5 (10.8)	< 0.0001	Mann-Whitney
Waist-Hip ratio risk	Low	9 (30%)	2 (6.7%)	< 0.0001	Chi-square*
	Moderate	18 (60%)	7 (23.3%)		
	High	3 (10%)	9 (30%)		
	Very high		12 (40%)		

*= considering low-moderate; ×-high-very high; BMI=body mass index

The periodontal aspects are presented in the Table 2. Pocket depth was deeper in the obese women, being the proportion of pockets between 4-5 mm higher in these women. Other periodontal aspects (calculus, BOP, and plaque index) were also bigger in the obese subjects and CAL did now show differences between groups. 

**TABLE 2:** Periodontal aspects of groups

	Median (interquartile deviation)		
Periodontal aspects	Normal weight	Obese	p (Mann-Whitney)
Probing pocket depth (mm)	1.9 (0.32)	2.3 (0.42)	0.0028
Pocket depth 0 to 3 mm (%)	98.8 (5)	91.7 (10.3)	< 0.01
Pocket depth 4 to 5 mm (%)	1.2 (4)	4.5 (8.6)	
Pocket depth more than 6 mm (%)	0 (0)	0 (0)	
			
Clinical Attachment Level (mm)	0 (0.02)	0 (0)	0.13
Calculus - n (%)	1 (4) 3.6% (14.3%)	4 (2.8) 14.3% (9.9%)	0.0105
BOP - n (%)	3 (5.5) 10.7% (19.7%)	8.5 (6.8) 30.4% (24.1%)	< 0.0001
Mean plaque index	1.3 (0.65)	1.8 (0.93)	< 0.0001

BOP=bleeding on probing

There were no differences between groups considering OHIP and its subscales, except for functional limitation, which was higher in obese subjects (Table 3).

**TABLE 3:** Oral Health Impact Profile (OHIP) and subscales presented according to the groups

	Median (interquartile deviation)		
	Normal weight	Obese	p (Mann-Whitney)
OHIP total score	1.5 (2.4)	2.2 (6.72)	0.22
Functional limitation	0 (0.37)	0.5 (0.51)	0.0254
Physical pain subscale	0.8 (1.24)	0.8 (2.41)	0.74
Psychological discomfort	0 (0.53)	0.2 (1.78)	0.24
Physical disability	0 (0.48)	0 (0.48)	0.75
Psychological disability	0 (0.6)	0 (1.45)	0.36
Social disability	0 (0)	0 (0)	0.29
Social handicap	0 (0)	0 (0)	0.86

**FIGURE 1:**
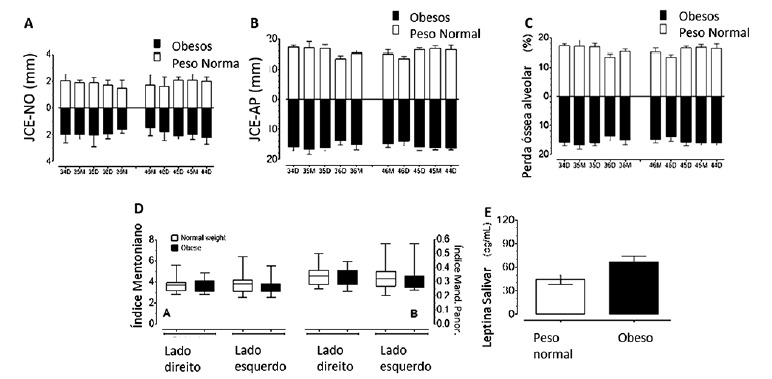
A) Median (bars) and interquartile deviation (whiskers) of CEJ-BL according to the groups and teeth aspects; B) median (bars) and interquartile deviation (whiskers) of CEJ-AP according to the groups and teeth aspects; C) median (bars) and interquartile deviation (whiskers) of alveolar bone loss according to the groups and teeth aspects; D) mental A and panoramic mandibular B indexes according to the groups and sides. Central line=median; boxes (1st and 2nd quartiles); whiskers=min and max values; E) salivary concentration (mean±SEM) of leptin according to the groups.

Figures 1A and 1B show the distance between the cement enamel junction to the bone level (CEJ-BL) and to the apex (CEJ-AP), respectively, according to the groups and aspects (mesial and distal) of the teeth observed. Figure 1C shows the alveolar bone loss according to the groups and teeth aspects. No differences (p>0.05) were observed between groups in any of the teeth aspects for CEJ-BL, CEJ-AP, and alveolar bone loss. 

*CEJ-BL=cement-enamel junction to bone level; CEJ-AP=cement-enamel junction to the apex

The trabecular patterns of the alveolar bone, according to the groups and sites of evaluation, are shown in Table 4. Normal-weight subjects showed higher proportion of dense bone trabeculae than obese subjects for both left (p=0.0053) and right (p=0.0017) sides of 1^st^ to 2^nd^ pre-molars’ measurements. However, the 2^nd^ pre-molar to 1^st^ molar measurement did not show significant differences (p>0.05) between groups in both left and right sides.

**TABLE 4:** Distribution of the scores of trabecular bone patterns

		Alveolar bone trabeculae - n (%)					
		Left side			Right side		
		Sparse (score 1)	Large (score 2)	Dense (score 3)	Sparse (score 1)	Large (score 2)	Dense (score 3)
1st to 2nd pre-molars	Normal weight	3 (10%)	4 (13.3%)	13 (43.3%)	3 (10%)	4 (13.3%)	13 (43.3%)
	Obese	7 (23.3%)	9 (30%)	4 (13.3%)	8 (26.7%)	9 (30%)	3 (10%)
2nd pre-molar to 1st molar	Normal weight	5 (16.7%)	4 (13.3%)	11 (36.7%)	6 (20%)	6 (20%)	8 (26.7%)
	Obese	9 (30%)	6 (20%)	5 (16.7%)	7 (23.3%)	8 (26.7%)	5 (16.7%)

Figure 1D shows the mental and panoramic mandibular indexes. No significant differences were found between groups for both indexes (p>0.05), irrespectively the side (left or right). In addition, only two (6.7%) of obese and one (3.35%) of the normal weight subjects presented osteopenia with semilunar defects.

The leptin concentration in saliva is shown in Figure 1E. Obese subjects showed higher (p=0.0261) concentrations of leptin than the normal-weight ones. 

Despite statistically significant (p<0.05), leptin concentrations showed weak (rS<0.4) and direct correlation with some of demographic characteristics of the patients. No correlation (p>0.05) was observed between leptin concentration and any of the periodontal aspects, OHIP (or its subscales), trabecular bone patterns, mental and panoramic mandibular indexes. 

In addition, the linear regression showed that the leptin concentration was dependent only on BMI (F, df=6.13, 59; p=0.016). The obtained model is presented in Table 5.

**TABLE 5:** Linear regression forward of leptin concentration

	Unstandardized Coefficients		Standardized coefficients		95% Confidence interval for B	
	B	S.E.	Beta	p	Lower	Upper
Constant	21.66	14.9	-	0.15	-8.1	51.4
BMI	0.97	0.39	0.31	0.0162	0.19	1.76

BMI=body mass index

## DISCUSSION

The present study was designed to evaluate possible association among salivary leptin concentrations, obesity, periodontal disease and some bone characteristics. The two groups observed in this study were distinct regarding all the anthropometric aspects, but height. The obese women clearly presented high values of the parameters most used to indicate morbid obesity condition, especially the waist-hip ratio, which is considered the most sensitive indicator for obesity^21^. 

In the present study, only women aging less than 35 years-old were included in both groups in order to remove the menopause bias from the study. Obesity patterns, distribution of body fat, and osteoporosis epidemiology differ between men and women, and between pre- and postmenopausal women^22^. 

Several studies are contradictory, since some showed positive association between obesity and increased bone mass, while others reported a negative association^7^ ^,^ ^9^. Since menopause could be a serious interference for the balance between obesity and bone mineral density, only premenopausal were observed in the present study. In this condition, obese women showed lower proportion of dense bone trabeculae than the non-obese ones, similar results of Salamat et al.^22^. Indeed, the relationship between obesity and skeleton is complex and not yet fully understood. Excessive visceral fat and fat mass have negative effects on bone health, being associated with low total bone mineral density and content^23^. Osteopontin is a protein expressed mainly in cells with multivariate effects on bone morphogenesis and remodeling. It has been related as one of the most overexpressed contributing to osteoporosis genes in the adipose tissue of obese subjects^24^. 

New evidences have proposed a link between glucose and bone metabolism. Soft tissues calcification expressing bone matrix proteins could be involved directly or indirectly in the regulation of glucose homeostasis, suggesting that skeletal tissues could contribute to the regulation of energy metabolism^11^.

In this study, a high proportion of sparse bone trabeculae in some teeth of the obese women were verified and, thus, these women could present high risk to progression of alveolar bone loss, similar to risk observed in women with osteoporosis^22^. Papakitsou et al.^25^ showed a lower rate of bone formation, measured by the serum concentration of type I collagen pro-peptide, suggesting that the production of new collagen is inhibited in obese women. However, in our study, no differences were found between obese and non-obese women regarding both mental and panoramic mandibular indexes. 

Leptin levels are elevated in the modern typical obesity as observed in the present and in other studies^26^ ^,^ ^27^ ^,^ ^28^. However, in the present study, salivary leptin concentrations were weakly associated with most of women anthropometric characteristics, such as age, waist-circumference, hip-circumference, waist-hip ratio and weight. The salivary leptin concentration was dependent only on BMI, suggesting it could be used to investigate metabolic risks in obese women.

Leptin is one of the many key-contributors to the development of cardiovascular disease, notably in the context of obesity and leptin levels can predict myocardial infarction^29^. It has been considered as a novel molecular link among obesity, chronic inflammation, and periodontal disease. The direct release of large quantities of IL-1 and TNF-α affect the teeth supporting-tissues, which induces loss of alveolar bone, cementum, and periodontal ligament and increases the progression of periodontal disease^30^ ^,^ ^31^. 

In the present study, the obese women presented deeper pocket and also more calculus, BOP and dental plaque than the normal-weight women. In addition, the loss of alveolar bone could be modulated, at least in part, by weight gain and obesity alone^32^. 

Other recent evidences have suggested the potential influence of the periodontal infection may also result in oral pain and tooth loss, which in turn result in decreased mastication function, resulting in several learning and memory illnesses^33^. 

The present study has some limitations, such the cross-sectional design allows investigating for association and not for causality, sample size was relatively small. Moreover, the lack of comparison with men does not allow infer whether gender-related distribution of body fat differently affects the alveolar bone loss measurement in men and women^23^. Finally, due to the retrospective design of the study, not all confounding factors have been considered. Sex hormone levels were measured by commercial assays, which lack the reliability presented by newer liquid chromatography tandem mass spectrometry methods. In addition, CTX is now considered a marker for bone resorption^20^ and it was not used in the present study. 

The variation in leptin concentrations observed among studies could be strongly influenced by many confounders, since obesity, periodontal and metabolic diseases have different and sometimes complimentary effects on its concentration. However, the salivary leptin concentration measured here was clearly dependent only in BMI have no influence on this hormone concentration on obese women.

## CONCLUSION

Obesity affects the periodontal conditions, the alveolar bone pattern, and the salivary leptin concentration. Salivary leptin concentration was influenced only by BMI.
